# Association Genetics in Plant Pathogens: Minding the Gap between the Natural Variation and the Molecular Function

**DOI:** 10.3389/fpls.2017.01301

**Published:** 2017-07-25

**Authors:** Anne Genissel, Johann Confais, Marc-Henri Lebrun, Lilian Gout

**Affiliations:** Unité Mixte de Recherche Bioger, Institut National de la Recherche Agronomique, AgroParisTech Institut des Sciences et Industries du Vivant et de L'environnement, Université Paris-Saclay Thiverval-Grignon, France

**Keywords:** plant–pathogen interaction, natural variation, GWAS, phenotype-genotype, linkage disequilibrium, functional validation

## Introduction

One of the main goal in phytopathology is to better understand the molecular basis of plant–pathogen co-evolution through the identification of effectors and effector targets that play a role in natural phenotypic variation. Fortunately, next generation sequencing (NGS)—which can measure genetic variation at hundreds of thousands of markers across a genome, including for non-model organisms—is now helping to reach this goal. Among all possible strategies using NGS data, we expect that genome-wide association studies (GWAS) have the most potential to revolutionize the field of phytopathology. In contrast to QTL mapping, GWAS use outbred populations to capture the standing genetic variation, thus characterizing the raw material for evolution. By examining the natural phenotypic and genetic variation, association mapping can elucidate the genetic basis underlying complex traits. In the two decades since association mapping successfully detected common variants for human complex diseases (Risch and Merikangas, 1996) and with the publication of the first successful GWAS in humans in 2005 (Klein et al., 2005), the number of published GWAS keeps increasing. Researchers in the field of plant pathogens are now embarking on GWAS, with the promise to open new frontiers of research.

## Association mapping is emerging in the field of phytopathology

The feasibility of association genetics (within candidate regions or genome-wide) for identifying traits related to plant–pathogen interactions, has been demonstrated for various species including both host plants and pathogens. Paving the way, a few recent studies detected significant associations targeting resistance or (a)virulence loci. The first GWAS targeting phenotypes related to plant–pathogen interactions were done in the model species *Arabidopsis (A.) thaliana*. Even for this selfing species with an extent of linkage disequilibrium (LD) over a distance of 20 kb, Atwell et al. (2010) demonstrated the power of GWAS. A total of 107 phenotypes were tested using a high density SNP map for 191 natural accessions. This study revealed significant associations for disease resistance genes against the bacteria *Pseudomonas (P.) syringae* (genes known to mediate pathogenic bacteria recognition such as *RPM1, RPS2*, and *RPS5*), and confirmed the contribution of these loci to the natural variation of resistance of *A. thaliana* against *P. synrigae*. Subsequent studies reported more bacterial resistance loci in *A. thaliana* (Nemri et al., 2010; Huard-Chauveau et al., 2013; Debieu et al., 2016). In addition, many other resistance genes against pathogens have been identified in agriculturally important plant species such as in maize (Olukolu et al., 2014; Li et al., 2016), soybean (Chang et al., 2016), rice (Yoshida et al., 2009), wheat (Gurung et al., 2014), and barley (Turuspekov et al., 2016). Despite the promise of GWAS, until now there has been few association studies on the pathogen side, and in addition the approaches used to identify candidate loci were suboptimal *[Xanthomonas campestris* (Guy et al., 2013); *Ralstonia solanacearum* (Pensec et al., 2015); *Heterobasidion annosum* (Dalman et al., 2013); *Fusarium graminearum* (Talas et al., 2016); *Parastagonospora nodorum* (Gao et al., 2016)].

Overall, these findings demonstrate that GWAS is a successful method in the field of plant–pathogen interactions. In addition, when conducted with a sufficiently high-density marker map single-gene resolution of GWAS reaches far beyond the resolution of QTL mapping.

## Association mapping in plant pathogens: lessons from human GWAS

Based upon the premise that the contribution of a genetic variant to the phenotype is expressed, association mapping measures the significance of the association between the phenotype and the genotype. A detailed discussion of the statistical aspects of GWAS is beyond the scope of this article, a thorough discussion can be found in Balding (2006). Apart from the complicated statistics related to GWAS, we advice to read a recent review from Power et al. (2017), which considers in details many key elements on the methodology for GWAS. We believe that these considerations have to be considered carefully for plant pathogen GWAS as well. We argue that among them, sample size and LD are the most crucial.

Sample size must be considered because greater statistical power is obtained when increasing the sample size (Long and Langley, 1999). However, a suitable sample size to detect common variants with large phenotypic effect is not necessarily the same between species. In fact, different sample sizes can achieve the same power due to differences in ploidy level, recombination rate, and genetic diversity between the species under study. In addition, when sample size is not very large (i.e., less than few hundreds of individuals), rare variants with a minor allele frequency <5% should be excluded from the study because significant rare alleles represented by very few individuals are often spurious.

When carefully examined, LD greatly helps to improve the power of the study. Pairwise LD, as measured in association studies, corresponds to the non-random association of alleles at two loci. It is usually estimated from the coefficient correlation R^2^ and Lewontin's D′ (Gaut and Long, 2003; also see Mangin et al. (2012) for corrected R^2^ by considering the sample structure). The measure of LD is fundamental to predict the resolution of association studies. Lastly, to clarify how many significant markers are dragged along with the putatively causal marker, LD also should be well described in genomic regions covering top significant markers. The worst-case scenario is when there is a group of significant markers in “complete” LD. In that situation, complementary sequencing approaches using larger sample size may help to identify among associated markers which one(s) is causal. Complementary sequencing approaches also are beneficial when performing GWAS using RAD (Restriction site associated DNA)-sequencing that partially surveys the genome. Indeed if the LD drops quickly between adjacent (but potentially distant) markers, additional sequencing should be performed to call more variants in the region and identify the ones with the lowest associated *P*-values.

GWAS are now emerging in phytopathology; however, the vast heterogeneity of species that are under study and the lack of a uniform design for all systems remains a challenge. Thus, thorough analyses of GWAS are necessary to positively impact the reliability of this approach in the field of phytopathology.

## Plant pathogen GWAS: the need for considering structural variants

Association genetics is commonly performed on SNPs called from sequencing data. SNPs represent the highest frequency variants in genomes; nonetheless, insertions, deletions, and other structural variants (e.g*.*, inversions, translocations within and between chromosomes) are not rare in micro-organisms and have been largely neglected so far. In our opinion, structural variants likely contribute to the potential of plant pathogen evolution and thus should be carefully examined. Hartmann et al. (2017) recently found that segmental deletion polymorphism within an effector gene is associated with virulence in the fungus *Zymoseptoria tritici* against wheat. Since structural variants can be identified using current genomic tools, we suggest structural variants need to be considered for GWAS.

## Interpreting GWAS and the search for causality

GWAS studies yield a number of significant SNPs that must be interpreted cautiously regarding the inference of causality. For example, a candidate SNP can be thought of as DNA variation at a given position, which contributes to a proportion of the natural phenotypic variation in the population. It is a good candidate (i.e., putatively causal) because we observe the most significant difference of disease symptoms between the two alleles at that position, within a given region. For instance, for a haploid species with two alleles “A” and “B” at the site, the phenotypic value varies the most significantly between the two groups “A” vs. “B.” Once we have this result, however, additional work to elucidate the molecular function associated with the SNP needs to be conducted to support causality (i.e., the allelic variation at the SNP truly causes the variation of the phenotype). This step, the so-called “functional validation” is commonly feasible in micro-organisms. This resource should be better exploited to examine fully the molecular function hidden behind a top significant marker.

The functional approach can be optimized depending on the nature of the putative causal variant. Different strategies may be undertaken if the variant is regulatory or coding. For a SNP in a coding region, mutant lines can be produced from the wild type by knockout, and complement mutants can be produced to rescue the phenotype. If a variant is regulatory and within or nearby a gene, we need to test whether it has an effect on the tempo and mode of expression of that gene, by looking at natural variation of the gene expression within the sample. When molecular tools are available to transform the species, a more tedious strategy can be attempted. Regardless of the nature of the variant, mutant lines using allele swapping (for bi- or polyallelic sites) can be constructed in several genetic backgrounds and phenotypic effects of each mutation can be quantified. We think this strategy should be prioritized for a better understanding on the molecular mechanisms associated with plant pathogen evolution. Using GWAS and reverse genetics one demonstration of the molecular co-evolutionary dynamics between host and pathogens was done (Huard-Chauveau et al., 2013; Wang et al., 2015). The identification of a pseudokinase (RKS1) for the resistance of *A. thaliana* against *X. campestris* pv. *campestris* by GWAS (Huard-Chauveau et al., 2013) was supported by complemented study from Wang et al. (2015) who elucidated the role of that pseudokinase in AvrAC effector recognition.

The use of different, interconnected approaches is clearly an advantage to validate the GWAS results. For example, QTL mapping with recombinant populations can be used to confirm a region (Nemri et al., 2010). This strategy is more applicable when recombinant populations with high-density marker map are available. Alternatively, independent association genetics validation is also a relevant strategy. Often neglected except for human GWA, replication of association mapping greatly contributes to elucidating causality, with the advantage of examining the natural genetic variation segregating within populations that have different recombination history. There is indeed a strong hint for causality if two independent association studies reveal the same top variant, even though the two replicate studies use different population samples.

## Conclusion

Examining the genotype-phenotype link may lead to an attractive list of genes useful for molecular studies in plant pathology and resistance management strategies. Yet, association genetics that aims at finding causal variants also provides the opportunity to address fundamental questions in evolutionary quantitative genetics. These questions are the following: (1) What molecular variants contribute the most to the standing phenotypic variation? (2) What is their phenotypic effect? (3) Are they regulatory or coding? (4) Are there many loci in action with small effects? (5) What molecular functions are associated with the phenotypic differences among individuals? (6) What evolutionary forces are maintaining this variation?

Importantly, GWA has limitations that may vary depending on the species and the phenotype under study and it is not trivial to perform a successful GWAS with good statistical power to detect causal variants. Even if molecular variants with large phenotypic effect are often discovered, certainly many more variants that contribute to the phenotypic variation remain to be found. The ability to detect these variants will require alternative strategies. We suggest that using very large sample sizes and combining population genomics with population transcriptomics and epigenetics should greatly contribute to elucidating the complex genotype-phenotype map in plant pathogen interactions.

## Author contributions

AG conceived and wrote the article with significant intellectual input from all authors: JC, ML, and LG.

### Conflict of interest statement

The authors declare that the research was conducted in the absence of any commercial or financial relationships that could be construed as a potential conflict of interest.
